# Characteristics of Sudden Bath-Related Death Investigated by Medical Examiners in Tokyo, Japan

**DOI:** 10.2188/jea.JE20140068

**Published:** 2015-02-05

**Authors:** Hideto Suzuki, Wakako Hikiji, Takanobu Tanifuji, Nobuyuki Abe, Tatsushige Fukunaga

**Affiliations:** Tokyo Medical Examiner’s Office, Tokyo Metropolitan Government, Tokyo, Japan

**Keywords:** bath-related death, sudden death, forensic autopsy, medical examiner

## Abstract

**Background:**

Sudden bath-related deaths occur frequently in Japan, particularly among elderly people. However, the precise mechanism of bath-related death remains uncertain, and effective prevention strategies have not been established.

**Methods:**

Cases of bath-related deaths (*n* = 3289) were selected from all cases handled by the Tokyo Medical Examiner’s Office from 2009 to 2011 (*N* = 41 336). The ages and occurrence dates were examined, and major autopsy findings, including toxicological analysis, were evaluated for the autopsied cases (*n* = 550).

**Results:**

Most cases occurred in individuals older than 60 years of age during winter. Analysis of autopsy findings revealed water inhalation signs in many cases (*n* = 435, 79.1%). Circulatory system diseases constituted more than half of the pathological findings regarding factors that may have contributed significantly to death (*n* = 300, 54.5%), and cardiac lesions were the most common pathological finding (*n* = 250, 45.5%). However, approximately one-third of the cases exhibited no remarkable pathological findings (*n* = 198, 36.0%). A quarter of all cases involved blood ethanol levels that exceeded 0.5 mg/mL (*n* = 140).

**Conclusions:**

The results suggested that drowning plays an important role in the final process of bath-related death. Circulatory system diseases may be the primary underlying pathology; however, there were variations in the medical histories and pathologies of cases of bath-related death. From a preventive perspective, family members should pay attention to elderly people with circulatory system diseases during bathing, particularly in winter. Additionally, the notion that ill or inebriated individuals should not take baths should be reinforced.

## INTRODUCTION

Many Japanese people relax by taking a bath. Unlike their European and American counterparts, the majority of Japanese people soak in standing water almost daily. Sudden death in a bathtub occurs relatively frequently in Japan, particularly among elderly people.^1^^–^^4^ The annual mortality rate for fatal drowning in Japan is higher than in other developed countries, and this is mainly attributable to bath-related deaths among elderly people.^4^^,^^5^ However, the actual number of deaths exceeds the official number of bath-related deaths because many bath-related deaths have been attributed to sudden natural causes such as ischemic heart disease on death certificates.^4^

A previous study showed that bath-related deaths typically occur during the winter months.^1^^–^^4^ It is believed that a rapid change in body temperature, attributable to large differences between the bath water temperature and the ambient temperature in the dressing room, is a critical factor that is capable of inducing sudden death, particularly in elderly people.^4^ However, the precise mechanism of bath-related deaths is unknown, and preventive strategies have not been established because the majority of bath-related deaths do not lead to autopsy in the current Japanese death investigation system.^1^ The need to gather objective evidence on autopsied cases has been emphasized^1^; however, there is a paucity of literature regarding autopsied cases of bath-related deaths.^4^

In the current Japanese death investigation system, the police usually determine whether an autopsy should be performed in areas where there is no medical examiner system.^6^^–^^8^ The police perform death scene investigation from a criminal standpoint and do not generally order an autopsy for bath-related deaths. In non-criminal cases, administrative autopsies are sometimes performed from a public health standpoint,^6^^–^^8^ but the number of administrative autopsies conducted is relatively low in areas without a medical examiner system. The medical examiner system has currently been implemented in only 5 cities in Japan, and Tokyo is the largest city with a medical examiner system.^8^^,^^9^

In this study, we investigated a large number of autopsied bath-related deaths in Tokyo, Japan. The study focuses on demographic and autopsy findings (eg, water inhalation and pathological findings of major viscera), including the results of toxicological analysis, to develop effective strategies for the prevention of bath-related death.

## METHODS

### Study sample

All sudden unexpected deaths in the special wards of metropolitan Tokyo, including those reported as sudden unexpected deaths from disease, non-disease-related causes, or unknown causes, are reported to the Tokyo Medical Examiner’s Office, and medical examiners perform postmortem examinations to determine the manner and cause of death. Medical examiners perform autopsies when the cause of death cannot be determined by medical history, course of illness, or situational or external investigations of the deceased.

In this study, documents of cases handled by the Tokyo Medical Examiner's Office from 2009 to 2011 (*N* = 41 336) were reviewed. We selected cases involving death that occurred in a bathtub, and cases in which death occurred out of water were excluded from the study. Documents available for review were death certificates, medical examiners’ reports on postmortem findings, and autopsy reports, which included toxicological analysis (if performed). The study sample consisted of 3289 cases (1702 men and 1587 women), which represents 8.0% of all cases handled by medical examiners during the study period. We examined the age, sex, occurrence date, and location where the death occurred for each case. Cases of death from drowning in rivers/seas were selected as controls (*n* = 413; 290 men and 123 women), and age and occurrence date were compared with the study sample. Data regarding the total population in the special wards of metropolitan Tokyo were reported by the Statistics Division Bureau of General Affairs of the Tokyo Metropolitan Government.^10^

### Autopsy findings

We examined the major autopsy findings of the autopsied cases (*n* = 652; autopsy rate: 19.8%). Cases involving people younger than 10 years and suicide were excluded because such cases were extremely rare in this study sample. In addition, criminal cases transferred to other facilities (ie, forensics departments of universities) for judicial autopsy and cases with severe putrefaction were excluded from the analysis. After excluding these cases, a total of 550 autopsied cases of bath-related death were analyzed.

We examined whether water inhalation signs were observed in each case. We defined “water inhalation signs” as a combination of findings suggesting death from drowning, such as froth in the air passage spaces, aqueous emphysema, aqueous pulmonary edema, and Paltauf’s spots. In addition, we listed the pathological findings that may have significantly contributed to death in each case (one finding for each case).

We examined aspects of cardiac pathology, including the extent of coronary artery stenosis and cardiomegaly, because our preliminary investigation showed that these features were present in a large majority of the cardiac pathology cases in bath-related deaths. Coronary artery stenosis greater than 75% was regarded as significant. We calculated normal heart weight (g) using body weight (kg) and height (cm) in each case, according to the formula reported by Hitosugi.^11^ Using calculated normal heart weight as a reference, the degree of cardiac hypertrophy was calculated in a manner similar to a previous study,^12^ and we regarded a degree of cardiac hypertrophy ≥20% as significant. Blood ethanol level was assessed using the value of n-propanol in each case, and blood ethanol levels that exceeded 0.5 mg/mL were regarded as significant.

We compared major autopsy findings and mean blood ethanol levels between cases exhibiting and not exhibiting water inhalation signs. In addition, we selected autopsied cases of death from drowning in rivers/seas (age ≥60 years, excluding suicidal cases, *n* = 87; 59 men and 28 women) as controls, and compared the proportions of cases with cardiac pathology between cases of death from drowning in rivers/seas and cases of bath-related death (age ≥60 years, *n* = 453; 274 men and 179 women).

### Statistical analysis

Intergroup comparisons were performed using the *χ*^2^ test for independence or the *t* test, where appropriate. The level of significance was set at *P* < 0.05.

### Ethical approval

The Ethics Committee of the Tokyo Medical Examiner’s Office approved the study protocol and use of data.

## RESULTS

### Demographic findings of bath-related deaths

**Figure 1.**
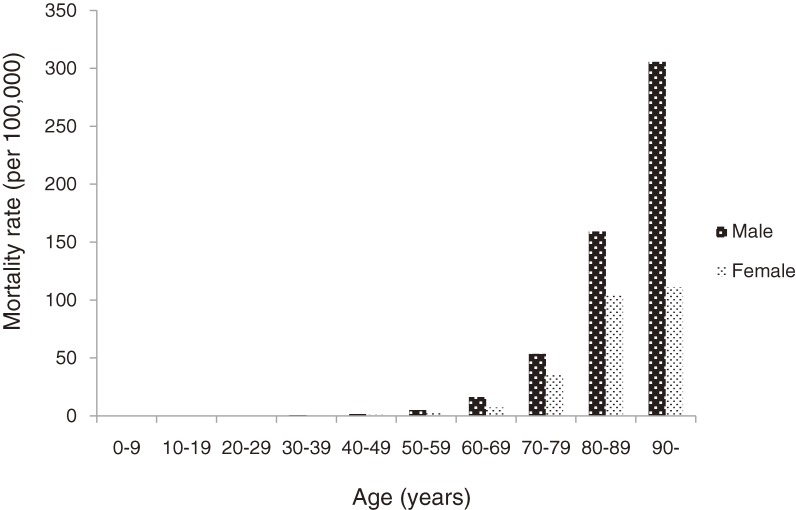
Age-specific mortality rates of bath-related deaths

Bath-related death was most frequently reported in individuals aged 80 to 89 years in both sexes (Table 1). In general, more than 90% of deaths in both sexes were in people older than 60 (92.5% of men and 95.7% of women). The age-specific mortality rates tended to increase with age in both sexes, and mortality rates in males were significantly higher than in females in all age groups aged ≥60 years (*P* < 0.01; Figure 1).

**Table 1.** Age distribution of bath-related deaths (*n* = 3289)

Age, years	Men			Women		
	
	Total cases (*n* = 1702)	Autopsied cases (*n* = 396)	Autopsy rate (%)	Total cases (*n* = 1587)	Autopsied cases (*n* = 256)	Autopsy rate (%)
0–9	3 (0.2%)	3 (0.8%)	100	1 (0.1%)	1 (0.4%)	100
10–19	2 (0.1%)	2 (0.5%)	100	0	0	
20–29	5 (0.3%)	5 (1.3%)	100	6 (0.4%)	4 (1.6%)	66.7
30–39	14 (0.8%)	13 (3.3%)	92.9	10 (0.6%)	7 (2.7%)	70.0
40–49	27 (1.6%)	20 (5.1%)	74.1	20 (1.3%)	13 (5.1%)	65.0
50–59	76 (4.5%)	39 (9.8%)	51.3	32 (2.0%)	22 (8.6%)	68.8
60–69	255 (15.0%)	126 (31.8%)	49.4	117 (7.4%)	52 (20.3%)	44.4
70–79	540 (31.7%)	115 (29.0%)	21.3	463 (29.2%)	90 (35.2%)	19.4
80–89	619 (36.4%)	68 (17.2%)	11.0	753 (47.4%)	58 (22.7%)	7.7
≥90	161 (9.5%)	5 (1.3%)	3.1	185 (11.7%)	9 (3.5%)	4.9

Parentheses indicate the proportion of cases to the total number in each group.

The most frequently autopsied cases were in men aged 60 to 69 years and in women aged 70 to 79 years (Table 1). The mean age of autopsied cases (67.4 years for men and 70.5 years for women) was significantly lower than that of non-autopsied cases (79.3 years for men and 81.4 years for women; *P* < 0.01). The overwhelming majority of bath-related deaths occurred in the deceased’s own residence (94.3%; Table 2).

**Table 2.** Settings of bath-related deaths (*n* = 3289)

Home	3102 (94.3%)
Communal bath	100 (3.0%)
Hotel	52 (1.6%)
Nursing home and hospital	27 (0.8%)
Others	8 (0.2%)

**Figure 2.**
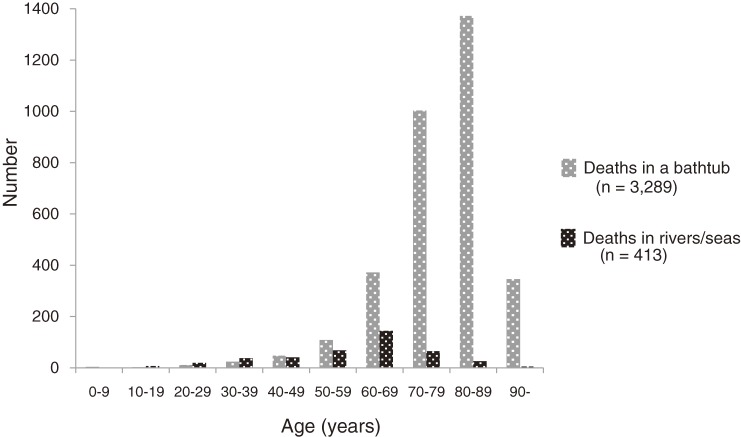
Age distributions of bath-related deaths and deaths in rivers/seas

**Figure 3.**
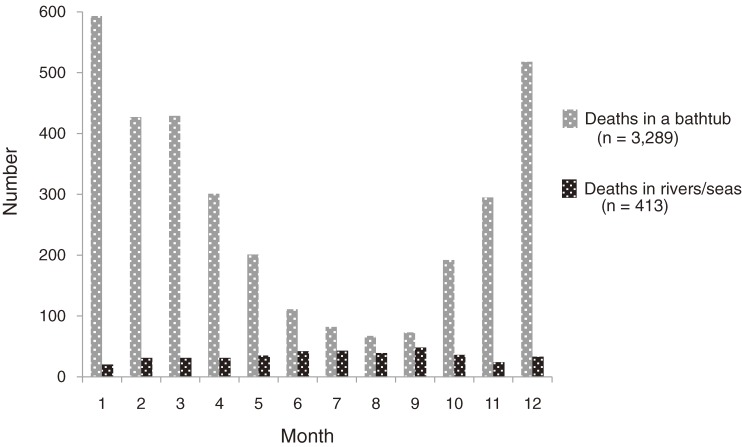
Number of bath-related deaths and deaths in rivers/seas according to month

The mean age of all cases of bath-related death (76.5 years for men and 79.6 years for women) was significantly higher than for cases of death from drowning in rivers/seas (57.1 years for men and 61.6 years for women; *P* < 0.01; Figure 2). A seasonal difference in occurrence dates was also evident. Death occurred more frequently in winter (Figure 3), and the number of deaths in winter months (from December through February; *n* = 1538) was 6.9 times higher than in the summer (from July to September; *n* = 222). The proportion of deaths occurring in winter (46.8%) was significantly greater among cases of bath-related death than among controls (20.3%; *P* < 0.01).

### Autopsy findings of bath-related deaths

Water inhalation signs were observed in a large majority of autopsied cases (*n* = 435, 79.1%; Table 3). There were no significant differences in the mean age or male/female ratio with respect to the presence of water inhalation signs.

**Table 3.** Comparison of major autopsy findings according to water inhalation signs

	Total cases (*n* = 550)	Water inhalation signs (+) (*n* = 435)	Water inhalation signs (−) (*n* = 115)
Circulatory disease	300 (54.5%)	234 (53.8%)	66 (57.4%)
Coronary artery stenosis and/or cardiomegaly	239 (43.5%)	188 (43.2%)	51 (44.3%)
Other heart disease (eg, valvular disease)	11 (2.0%)	5 (1.1%)	6 (5.2%)**
Cerebral artery sclerosis and/or cerebral infarction	29 (5.3%)	29 (6.7%)	0**
Cerebral hemorrhage	15 (2.7%)	9 (2.1%)	6 (5.2%)
Other circulatory disease	6 (1.1%)	3 (0.7%)	3 (2.6%)
Non-circulatory system disease	47 (8.5%)	28 (6.4%)	19 (16.5%)**
Respiratory disease	13 (2.4%)	6 (1.4%)	7 (6.1%)**
Digestive disease	4 (0.7%)	1 (0.2%)	3 (2.6%)**
Neoplasms	13 (2.4%)	8 (1.8%)	5 (4.3%)
Endocrine, nutritional and metabolic diseases	7 (1.3%)	5 (1.1%)	2 (1.7%)
Nervous disease	5 (0.9%)	5 (1.1%)	0
Genitourinary system	5 (0.9%)	3 (0.7%)	2 (1.7%)
Trauma	5 (0.9%)	4 (0.9%)	1 (0.9%)
No pathological findings	198 (36.0%)	169 (38.9%)	29 (25.2%)**
History of epilepsy	13 (2.4%)	12 (2.8%)	1 (0.9%)
Psychotropic drug poisoning	8 (1.5%)	7 (1.6%)	1 (0.9%)
Others	177 (32.2%)	150 (34.5%)	27 (23.5%)*
Blood ethanol level exceeded 0.5 mg/mL	140 (25.5%)	121 (27.8%)	19 (16.5%)*
Mean blood ethanol level (mg/mL)	0.44	0.48	0.28*

Percentages indicate the proportion of each finding to the total number of cases in each group.

**P < 0.01, *P < 0.05.

Circulatory system diseases constituted more than half of the pathological findings that could have contributed significantly to death (*n* = 300, 54.5%; Table 3). Sub-classification of circulatory system diseases showed that coronary artery stenosis and/or cardiomegaly was the most common pathological finding (*n* = 239, 43.5%; Table 3). The second-most common autopsy finding was atherosclerotic lesion of cerebral arteries (*n* = 29, 5.3%; Table 3).

Pathologies not related to the circulatory system were also observed (*n* = 47, 8.5%). Non-circulatory system pathologies were divided into the following 5 categories: central nervous system pathology (eg, advanced brain tumor and Wernicke encephalopathy; *n* = 12), infection (eg, pneumonia and pyelonephritis; *n* = 10), metabolic disturbance (eg, severe dehydration and diabetic/alcoholic ketoacidosis; *n* = 10), pathology affecting the heart (eg, cor pulmonale and chronic thyroiditis with severe cardiomegaly; *n* = 9), and others (*n* = 6; data not shown). There were 5 cases of traumatic lesion (Table 3), which included 3 cases of acute intracranial injury.

Approximately one-third of cases exhibited no remarkable pathological findings (*n* = 198, 36.0%), including 13 cases of epilepsy and 8 cases of psychotropic drug poisoning (ie, major/minor tranquilizer; Table 3). Among the 8 cases of psychotropic drug poisoning, 7 had psychiatric illness (4 and 3 cases with schizophrenia and depression, respectively); drug abuse was not apparent among these cases. Seventy-six of the remaining 177 of these cases exhibited blood ethanol levels exceeding 0.5 mg/mL; however, blood ethanol levels were not markedly high in 101 cases (18.4%, data not shown).

There was no significant difference in the proportion of cases involving circulatory system pathology with respect to the presence of water inhalation signs (present in 53.8% of cases and absent in 57.4%; Table 3). On the other hand, the proportion of cases with non-circulatory system pathology was significantly greater among cases without water inhalation signs. Furthermore, the proportions of cases with no pathological findings and markedly high blood ethanol levels were significantly greater among cases with water inhalation signs than without (Table 3). The proportion of cases with cardiac pathology was greater among cases of bath-related death (56.2% of men and 78.8% of women) than among other drowning cases (35.6% of men and 50.0% of women; *P* < 0.01; Table 4).

**Table 4.** Comparison of cardiac pathology between bath-related deaths and other drowning cases

	Men		Women	
	
	Bath-related death (*n* = 274)	Other drowning cases (*n* = 59)	Bath-related death (*n* = 179)	Other drowning cases (*n* = 28)
Cardiac pathology	154 (56.2%)	21 (35.6%)**	141 (78.8%)	14 (50%)**
Others	120 (43.8%)	38 (64.4%)	38 (21.2%)	14 (50%)

Percentages indicate the proportion of each finding to the total number of cases in each group.

**P < 0.01.

## DISCUSSION

The age distribution and seasonal variation in rate of bath-related deaths are distinct from those of other drowning deaths. Bathing among elderly people in winter appeared to be a major potential target for the prevention of bath-related sudden death. Water inhalation sign was observed in a great majority of the cases of bath-related death, which suggests that drowning is strongly associated with the final process of death. Therefore, finding victims before the completion of drowning may be crucial to prevent bath-related death.

Cardiac lesions were the most common pathological findings that may have contributed significantly to death, irrespective of the presence of water inhalation signs. In addition, the proportion of cases with cardiac pathology was greater among cases of bath-related death than among other drowning cases. These findings suggest that cardiac lesion may be strongly associated with bath-related death. Several physiological studies have demonstrated the occurrence of significant ECG changes during bathing among elderly people with underlying cardiac diseases. Igarashi reported that 21 of 60 patients who were diagnosed with angina pectoris showed ECG changes (ischemic change or arrhythmia) during bathing.^13^ Other studies have shown that the double product (systolic blood pressure × heart rate), which reflects myocardial oxygen consumption, increased in elderly people immediately upon immersion, while it remained constant in younger people.^14^ These observations support the notion that underlying cardiac diseases in elderly people are risk factors of death in the bathtub.

It has also been reported that there is a clear seasonal trend in acute cardiovascular events, with the highest incidence occurring during the cold winter months.^15^^,^^16^ In addition, the mortality rates of cardiac diseases in all age groups (≥60 years) were significantly higher in males than in females in the Tokyo Metropolitan area during the study period.^10^^,^^17^^–^^19^ Seasonal variation and sex differences in the mortality rate of bath-related death may be strongly influenced by the occurrence of cardiovascular events in the bathtub.

The second-most common autopsy finding was atherosclerotic lesions in cerebral arteries. Decreases in systolic blood pressure during bathing were shown in a previous experiment.^2^ Nagasawa et al speculated that hypotensive syncope, as a consequence of a decrease in the sympathetic tone that develops approximately 4 minutes after immersion, may cause sudden death by drowning during bathing in hot water.^14^ Underlying atherosclerotic lesions in cerebral arteries, together with a decrease in blood pressure, may reduce the blood flow to the central nervous system enough to cause loss of consciousness, resulting in drowning.

Even though the number of cases was small, diseases other than those of the circulatory system were also observed. In addition to having a direct effect on the heart and central nervous system (eg, cor pulmonale and brain tumors), subsequent or coexisting dehydration may play an important role in circulatory failure in cases such as infectious disease and ketoacidosis.

Around one third of the cases showed no remarkable pathological findings. The proportions of cases with no pathological findings and blood ethanol levels >0.5 mg/mL (as well as mean blood ethanol level) were both higher in cases with water inhalation signs, suggesting alcohol intake may be a crucial factor in cases with no remarkable autopsy findings besides drowning. In addition to reducing neural cell activity, acute alcohol consumption can cause atrial fibrillation in patients both with and without heart disease.^20^ These effects of alcohol may contribute to bath-related death. On the contrary, no remarkable pathological findings, drinking, drug use, or relevant past medical history (eg, epilepsy) were observed in 18.4% of cases. Factors that are difficult to prove in autopsies, such as neuromediated syncope and heatstroke^21^ induced by hot bath water, may be involved in these deaths; however, further studies are needed to elucidate the mechanisms of morphologically or toxicologically negative cases.

From a preventive perspective, more than one quarter of the deaths (eg, those related to alcohol intoxication) may have been avoided. It should be highlighted that inebriated or ill (eg, dehydrated) people should not bathe. In addition, family members should pay attention to elderly people who have circulatory diseases during bathing, particularly in winter.

This study has several limitations. First, a limited number of autopsied cases were analyzed (*n* = 550, representing 16.7% of total bath-related deaths in the study sample), and the age distribution of the autopsied cases was smaller than that of the total population of bath-related deaths (Table 1). In general, elderly victims in their late 80s and 90s do not undergo autopsy, because the majority have at least some medical history (eg, ischemic heart disease) to which the cause of sudden death can be attributed. Second, interpretation of water inhalation signs is not simple. Signs of water inhalation may be absent, even in cases of drowning, because a small amount of water can stimulate laryngeal spasm mediated by the vagal reflex. This may lead to an underestimation of the cases of drowning.

In conclusion, this study revealed that drowning may play a crucial role in the final process of bath-related sudden death. Although unexpected heart attack may be the most common underlying cause, the medical histories and pathological findings of cases of bath-related death vary. Preventive strategies include paying special attention to elderly people who have circulatory diseases during bathing and informing the public that ill or inebriated individuals should not bathe without supervision. Further accumulation of autopsy data, in addition to the study of physiological pathways relating bathing to sudden death, may elucidate detailed mechanisms of bath-related death in the future.
